# Incubation of canine dermal fibroblasts with serum from dogs with atopic dermatitis activates extracellular matrix signalling and represses oxidative phosphorylation

**DOI:** 10.1007/s11259-022-09947-y

**Published:** 2022-06-04

**Authors:** Monica Colitti, Bruno Stefanon, Misa Sandri, Danilo Licastro

**Affiliations:** 1 Departement of AgroFood, Environmental and Animal Science, University of Udine, via delle Scienze 206, 33100 Udine, Italy; 2 AREA Science Park, Padriciano, 99, 34149 Trieste, Italy

**Keywords:** Dermal fibroblast, Atopic dermatitis, Gene expression, *Canis lupus familiaris*

## Abstract

The aim of this study was to investigate the effects on gene expression in canine fibroblasts after incubation with a medium enriched with atopic dermatitis canine serum (CAD) compared with healthy canine serum (CTRL) and fetal bovine serum (FBS). Differential Expression and Pathway analysis (iDEP94) in R package (v0.92) was used to identify differentially expressed genes (DEGs) with a False Discovery Rate of 0.01. DEGs from fibroblasts incubated with CAD serum were significantly upregulated and enriched in the extracellular matrix (ECM) and focal adhesion signalling but downregulated in the oxidative phosphorylation pathway. Genes involved in profibrotic processes, such as TGFB1, INHBA, ERK1/2, and the downward regulated genes (collagens and integrins), were significantly upregulated after fibroblasts were exposed to CAD serum. The observed downregulation of genes involved in oxidative phosphorylation suggests metabolic dysregulation toward a myofibroblast phenotype responsible for fibrosis. No differences were found when comparing CTRL with FBS. The DEGs identified in fibroblasts incubated with CAD serum suggest activation of signalling pathways involved in gradual differentiation through a myofibroblast precursors that represent the onset of fibrosis. Molecular and metabolic knowledge of fibroblast changes can be used to identify biomarkers of the disease and new potential pharmacological targets.

**Supplementary Information:**

The online version contains supplementary material available at 10.1007/s11259-022-09947-y.

## Introduction

Atopic dermatitis in dogs (CAD) is a common, multifaceted, allergic skin condition that causes chronic pruritus. CAD often results in skin lesions such as erythema, hyperpigmentation, and lichenification. According to Olivry et al. (2014), CAD is often associated with the presence of IgE-specific allergens, but other studies have been shown that is not always IgE-mediated (De Boer 2004). Canine Atopic Dermatitis Extent and Severity Index (CADESI-4), is a scale to grade skin lesions created by scoring 20 different body sites typically affected in atopic dogs. For each site, 3 lesions (erythema, lichenification, and alopecia/excoriation) are scored on a scale of 0 to 3. However, CAD cannot be explained by a single mechanism (Bakker et al. 2021) and efforts are needed to better understand the pathogenesis at biomolecular level. CAD shares with human pathogenesis clinical features (Bizikova et al. 2015) and treatments (Olivry et al. 2015), and for this reason the dog is considered a model animal for translational studies (Martel et al. 2017).

Studies on the molecular mechanisms of CAD have shown that fillagrin (Kanda et al. 2013) and beta-defensin (van Damme et al. 2009) are key proteins for skin integrity. Other transcriptome approaches have revealed clusters of monocyte chemotactic, IL1 family, keratin (Plager et al. 2012), inflammation/immunology, transport and regulation, and barrier formation genes (Merryman-Simpson et al. 2008). Tengvall et al. (2020) found that genes involved in immune activation and in the inflammatory cascade are differentially expressed in dogs with CAD compared with healthy subjects. Several factors are likely responsible for the researchers’ different results, including the dog’s genetic predisposition, coat and skin, age, diet, and environmental factors, as well as variability in CAD lesions.

In vitro cell studies are an alternative way to investigate the molecular mechanism potentially involved in the onset of CAD, although results have limitations given the complexity of the onset and progression of the disease. Fibroblasts are an important component of connective tissue and participate in the regulation of the inflammatory cascade by producing inflammatory cytokines in response to stimuli (Tracy et al. 2016). Prolonged chronic inflammation, due to persistent infections, tissue injuries and allergic responses, leads to a switch of fibroblast in myofibroblast phenotype with the formation of stress fibers, the production of excess extracellular matrix (ECM) structural proteins and activation of fibrotic processes and cellular proliferation (Wynn 2008; Kendall and Feghali-Bostwick 2014). The activation is promoted by Transforming Growth Factor-β1 (TGFB1) and fibronectin containing extra domain A (EDA-FN) (Zent and Guo, 2018). Among these factors, activin A, which belongs to the TGFβ superfamily, plays a crucial role in wound healing and inflammation and is considered to be the link between the inflammatory process and the fibrotic response in skin diseases (Antsiferova and Werner 2012). Fibroblasts have been used as a non-invasive model to study the molecular mechanisms of skin response after exposure to oxidative stress, antioxidants (Pomari et al. 2013), interleukin 1β (IL-1β) (Kitanaka et al. 2019), and to unravel their role in the development of atopic dermatitis (Berroth et al. 2013; He et al. 2020; Savinko et al. 2012). In these studies, the medium was enriched with a specific compound, but this approach does not consider the complex interaction that from the simultaneous presence of multiple nutrients, growth factors, and regulatory components. The potential of conditioning culture media with serum was developed as a model to study in vitro the response of specific target cells in humans (Patel et al. 2019). The addition of serum collected from groups of individuals under different conditions to the culture medium provides an ex vivo method of conditioning the medium and modulates the in vitro cell response (Carson et al. 2018; Chua et al. 2007; Josh et al. 2012). In this approach, cells are exposed to a complex of compounds, nutrients, and growth factors that more closely resembles a real situation.

The aim of this study was to investigate gene expression using ex vivo canine serum to condition the medium and regulate gene expression of canine fibroblasts in vitro. For the study, the fetal bovine serum (FBS) in the medium was substituted with a pool of serum sampled from healthy dogs (CTRL), or a pool of serum sampled from dogs with clinical atopic dermatitis (CAD).

## Material and methods

### Selection of subjects

This study compared the effect of serum of dogs with and without CAD on cultured dermal fibroblasts. Client-owned dogs suffering from CAD were selected for the study based on a history of non-seasonal pruritic skin disease for at least 6 months with any type of lesion phenotype. At the first visit, dogs were without oral or topical drug treatment for at least 8 weeks. Moreover, skin parasites were excluded by means of skin scraping and elimination diet was performed to exclude food allergy. The CADESI-4 score (Olivry et al. 2014) was evaluated by clinicians and only dogs with on average score of 47.0 ± 3.7 were included in the study (Table 1). During the first visit, blood sample was collected for routine laboratory analysis consisting of blood count, hemocytometry, and biochemistry. Owners were asked to allow the collection of a subsample of the leftover blood for this study. According to the final diagnosis obtained by dermatological veterinary, only the pooled serum of dogs affected of CAD (n = 10) was later used in the in vitro study. Based on veterinary diagnosis, the healthy dogs (n = 10), owned by the clients, were selected and their sera were pooled (CTRL). These healthy dogs were without signs of CAD and current allergy from at least 6 months. In addition, all dogs were regularly vaccinated, free of heartworms, and had not been treated with antibiotics, corticosteroids, or other medications for at least 8 weeks.

**Table 1:** Breed, sex and age of healthy dogs (CTRL) and with atopic dermatitis (CAD) enrolled in the study

Breed	Sex	Age, (years)	Group	CADESI-4 (Score)
Boxer	MI	4	CTRL	healthy
German Shepherd	MI	5	CTRL	healthy
Australian Shepherd	MI	3	CTRL	healthy
German Shepherd	FI	3	CTRL	healthy
Golden	MI	6	CTRL	healthy
Flat Coated Retriever	FI	5	CTRL	healthy
Labrador	FI	4	CTRL	healthy
Mongroel	MI	7	CTRL	healthy
Labrador	FI	4	CTRL	healthy
Akita American	MI	3	CTRL	healthy
Labrador	MI	7	CAD	45
Dogo Argentino	MI	6	CAD	47
Carlino	FI	3	CAD	50
Mongroel	MI	5	CAD	40
Labrador	MI	6	CAD	48
Spinone Italian	FI	4	CAD	46
Westhighland Terrier	MC	6	CAD	51
Flat Coated Retriever	FI	4	CAD	47
Boxer	MI	5	CAD	43
Bulldog English	MI	3	CAD	52

*MI* male

*FI* female

*MC* male castrated

CADESI-4 Score (Olivry et al. 2014)

All animals in this study were the property of a responsible adult pet owner who gave informed consent for his or her pet to participate in the study.

### Cell culture

Canine immortalized dermal fibroblast-hTERT (ABM, Vancouver, Canada) were cultured in Dulbecco’s modified Eagle’s medium (DMEM), 2 mM l-glutamine, 100 U/ml penicillin and 100 μg/ml streptomycin (Pomari et al. 2013). FBS in the medium was substituted with a pool of CTRL serum, or with a pool of CAD serum.

All reagents were purchased from Euroclone (Pero, Milan, Italy). Cells were maintained in humidified air with 5% CO2 at 37 °C. Cells were grown to approximately 70–90% confluence and serum free DMEM was used for cell treatment.

Confluent dermal fibroblasts (1 × 10^4^ cells/well) were seeded on 96 well tissue culture plate and left to grow for 24 hours. Viability of dermal fibroblasts was measured by the 3-(4,5- dimethylthiazol-2-yl)-2,5-diphenyltetrazolium bromide (MTT) colorimetric assay. MTT test was performed on cells incubated for 24 h in a FBS at increasing doses 1.25%, 2.50%, 3.75%, 5.00% and in free medium (negative control) that was used as blank. Dermal fibroblasts were also incubated for 24 h with different percentages (1.25%, 2.50%, 3.75%, 5.00%) of serum pools from dogs without, or with CAD. The values from FBS were used as references to calculate percentage of viability. At the end of treatments 20 μl of MTT reagent (3-(4,5-dimethylthiazol-2-yl)-2,5-diphenyltetrazolium bromide) was added for 3 h. For each pool the test was conducted in triplicate and replicated two times (n = 6). Next, the mixture in each well was removed, and formazan crystals formed were dissolved in 100 μl of dimethyl sulfoxide (DMSO). Optical density of the mixture was measured in Spark multiplate reader at 550 nm. (Tecan, CH).

### RNA extraction and sequencing

For the gene expression study, RNA was extracted from cells incubated for 24 h with FBS, with CTRL or CAD serum at a dose of 50%. Briefly, after treatments, medium was removed from Petri dishes (2 technical replicates), 1 ml/10 cm^2^, cells were washed with PBS twice and total RNA was extracted using RNAeasy plus mini kit (Qiagen, Milan, Italy), according to the manufacturer’s instructions. RNA was quantified using a spectrophotometer (NanoDrop 1000 Spectrophotometer, ThermoScientific, Wilmington, Delaware) and the purity of RNA samples ranged between 1.8 and 1.9. Further, RNAs were assigned the RNA integrity number (RIN) score by the Agilent 2100 Bioanalyzer (Agilent Technologies, United States) (Schroeder et al. 2006). All samples had an high quality RIN and were processed into sequencing libraries using Illumina HiSeq2000 platform (http://www.illumina.com/systems/genome analyzer) (ESM_1).

The RNA sequencing data were deposited in the NCBI sequence Read Archive (SRA) under accession number of PRJNA803064.

### Data processing

The RNA raw sequences were trimmed to remove adapter sequence and the resulted unique tags were counted. Each unique sequence (tag) was aligned to NCBI_Assembly: GCF_000002285.5 genome to identify read correspondences to RNA using STAR software (Dobin et al. 2013) and a table of counts of each RNA in each sample was generated.

Raw counts were uploaded in Differential Expression and Pathway analysis (iDEP94) R package (v0.92) that is a web-based tool available at http://ge-lab.org/idep/ (Ge et al. 2018). In the preprocessing step, genes expressed at very low level across samples were filter out and genes expressed with a minimum of 0.5 counts per millions (CPM) in one library were further analyzed. To reduce the variability and normalized count data, EdgeR log2(CPM + c), with pseudocount c = 4 transformation, was chosen. Next, DESeq2 package in R language was used to identify differentially expressed genes (DEG) among treatments using a threshold of false discovery rate (FDR) < 0.01 and fold-change > |1.5|. The heatmaps, principal component analysis (PCA), k-means cluster and enrichment analyses were also performed in iDEP93.

Gene set enrichment analysis to determine the shared biological functions of differentially regulated genes based on significant GO terms (Ashburner et al. 2000) and Kyoto Encyclopedia of Genes and Genomes (KEGG) pathway (Kanehisa and Goto 2000) analyses were completed.

### Statistical analysis

Kruskall-Wallis nonparametric test was used to analyse statistical differences in cell viability between treatments (FBS, CTRL and CAD) within each dose (1.25%, 2.50%, 3.75%, 5.00%) with XLSTAT (Addinsoft 2020).

## Results

### Cell viability

To analyze the effect of doses in different serum pool exposure, cell viability assay was performed by treating dermal fibroblasts with increasing doses (1.25%, 2.50%, 3.75% and 5.00%) of CTRL and CAD sera for 24 h. After subtracting the blank signal (free medium) from the absorbance value, cell viability was calculated as the % compared to FBS at the same doses. The viability remained always around 80%, although significantly differences were observed after exposure at dose 1.25% between CAD and FBS (*P <* 0.001) and between CAD and CTRL (*P <* 0.05). The pairwise comparison showed significant differences at 3.75% between CTRL and FBS (*P <* 0.001). Both CTRL and CAD at dose 5.00% showed a significantly lower viability in comparison to FBS for *P <* 0.001 (ESM_1). No significant differences were observed between treatments at 2.50% dose that therefore was chosen for subsequent analyses.

#### RNAseq analysis

**Fig. 1:**
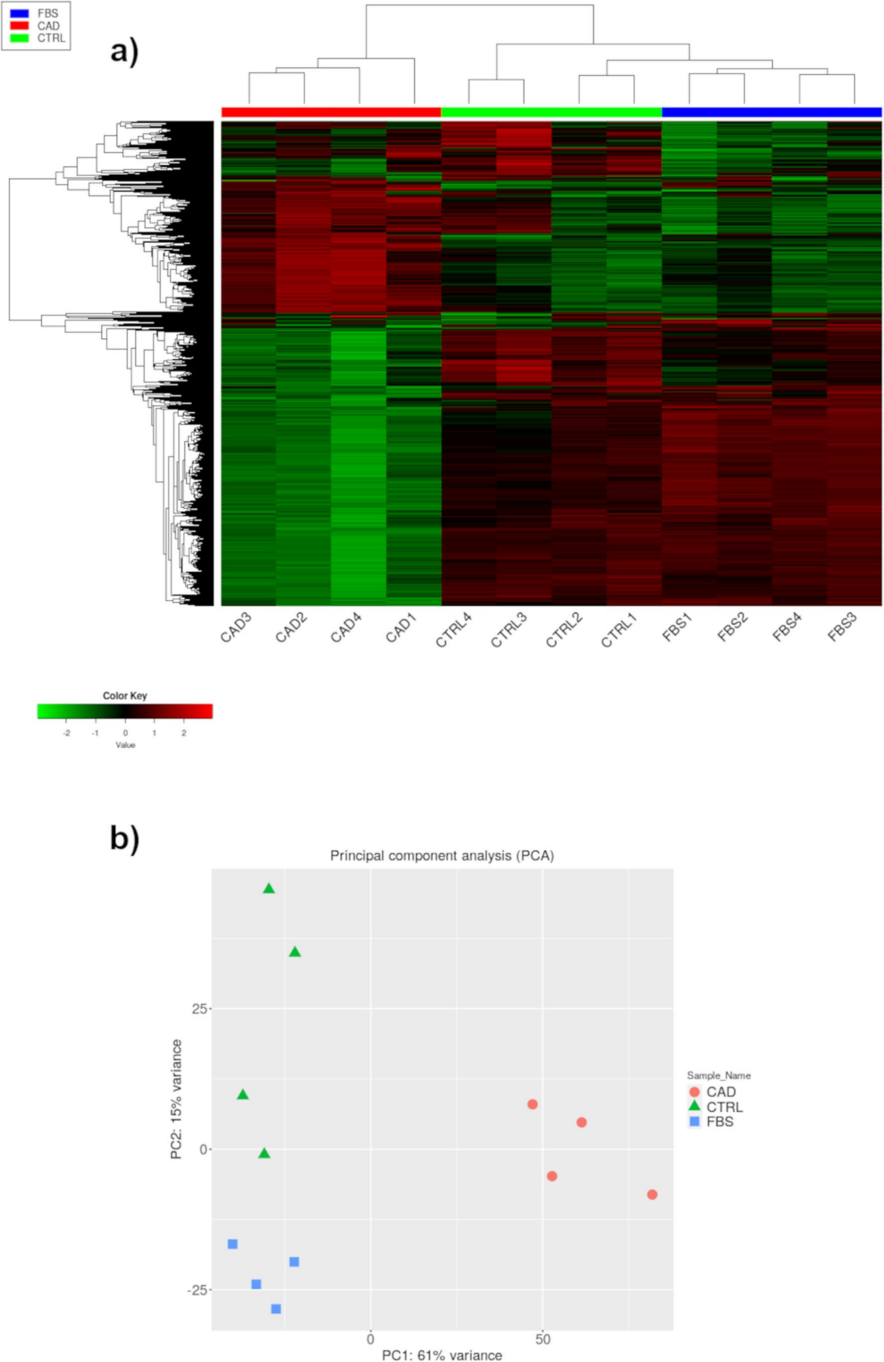
**a** Hierarchical clustering heatmap of the expression profiles of 2000 genes expressed in dermal fibroblasts from dog incubated with medium conditioned with fetal bovine serum (FBS), with serum of healthy dogs (CTRL) or with serum of dogs with atopic dermatitis (CAD). The colored bars above the heatmap indicate each treatment. The color key indicates z-score and displays the relative expression levels: green lowest expression; black intermediate expression; red highest expression. **b** Principal component analysis of fibroblast samples subjected to different treatments and to RNAseq. The results indicate that the transcriptome data are of good quality as the quadruplicate samples are clustered together according to treatment conditions. The CTRL and FBS serum samples were closely related in terms of their transcriptional patterns. Numbers from 1 to 4 indicate the 4 replicates within the CTRL FBS and CAD treatments. Green: gene significantly downregulated for *P <* 0.01; Red: gene significantly upregulated for *P <* 0.01

After removal of low-quantity reads, the final mapping rate of filtered transcript reads were 70.6%. At the initial analysis of the RNAseq results, hierarchical clustering was performed indicating the difference in 2000 genes and showing that the transcriptome data was well-clustered depending on treatments (Fig. 1a). Furthermore, principal component analysis (PCA) was performed to show the overall variability in the expression profile of the samples and treatments. There was a clear separation between the CAD and CTRL and FBS treatments along the first principal component (Fig. 1b), explaining 61% of the variance, with less differences along the second component (15% of total variance).

#### Identification of DEGs

Genes that were differentially expressed between the analyzed sample groups were selected, based on a fold change of > |1.5| and an adjusted p value of <0.01. The results of this selection are presented in the form of volcano plots in ESM_2.

**Fig. 2:**
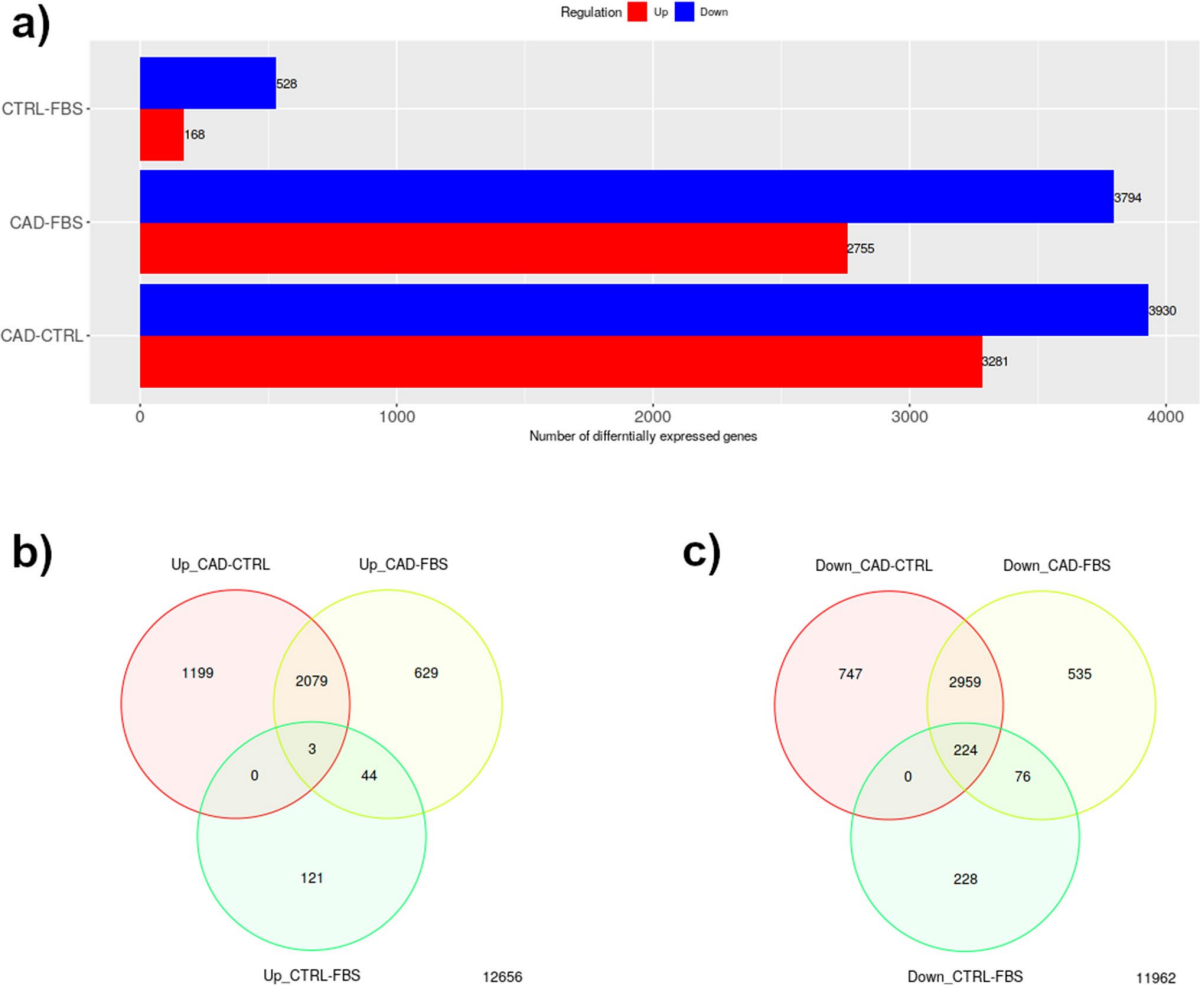
Number of differentially expressed genes for *P <* 0.01 and with | log2 fold change| > 1.5. Gene expressions were measured in canine dermal fibroblasts incubated in vitro with a pool of serum from healthy dogs (CTRL), with a fetal bovine serum (FBS) and with serum from dogs with atopic dermatitis (CAD). **a** Number of differentially expressed genes for each comparison. **b** Three-way Venn diagram illustrating that 3 overlapping transcripts were consistently upregulated in the serum culture relative to the condition (FDR < 0.01 and | log2 fold change| > 1.5) among treatments. **c** Three-way Venn diagram illustrating that 224 overlapping transcripts were consistently downregulated in the serum culture relative to the condition (FDR < 0.01 and | log2 fold change| > 1.5) among treatments

iDEP94 expression analysis identified significantly (*P <* 0.01) 528 upregulated genes between CRTL vs. FBS, 3794 upregulated genes between CAD vs. FBS and 3281 upregulated genes between CAD vs. CTRL, respectively (Fig. 2a). Significantly (*P <* 0.01) downregulated genes were 168 between CRTL vs. FBS, 2755 between CAD vs. FBS and 3281 between CAD vs. CTRL. Further analysis of these DEGs by using Venn diagram revealed that 3 and 224 common transcripts were consistently up and downregulated (FDR < 0.01 and |fold change| > 1.5), regardless of treatments (Fig. 2b and ESM_4).

#### Functional enrichment analysis

To further investigate the function of the DEGs, GO term enrichment analysis was conducted. The DEGs were significantly enriched in biological processes (BP), molecular function (MF) and cellular component (CC).

**Fig. 3:**
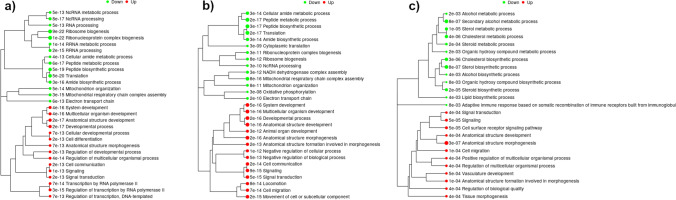
Biological process of GO enrichment analysis of DEGs from canine fibroblasts. A hierarchical clustering tree was constructed measuring the distance among the terms based on the percentage of overlapped genes. **a** Comparison between cells incubated with serum of dogs suffering from canine atopic dermatitis (CAD) and with medium enriched with serum from healthy dogs (CTRL); **b** Comparison of cells incubated with serum from dogs suffering from atopic dermatitis (CAD) and with medium enriched with fetal bovine serum (FBS). **c** Comparison of cells incubated with medium enriched with serum from healthy dogs (CTRL) and with medium enriched with fetal bovine serum (FBS)

Significantly enriched GO BP terms were 30 for the CAD_CTRL comparison, 29 for the CAD_FBS comparison and 25 for CTRL_FBS comparison (Fig. 3 and ESM 5). Of note, the number of DEGs within each term was much higher in the CAD_CTRL and CAD_FBS comparisons than in the CTRL_FBS comparison and this was also more evident when the same term was considered, as in the case of ‘Anatomical structure morphogenesis and Signaling’ (ESM_5). Interestingly, genes involved in fibrosis such as TGFB1, Activin A (INHBA), Activin A Receptor Type 1B (ACVR1B), SMAD Family Member 1 (SMAD1), Collagen Type I Alpha 2 Chain (COL1A2), Collagen Type IV Alpha 1 Chain (COL4A1), Collagen Type IV Alpha 2 Chain (COL4A2), Fibronectin 1 (FN1), Integrin Subunit Beta 3 (ITGB3), Integrin Subunit Alpha 2b (ITGA2B), lysyl oxidase-like proteins (LOXL1, LOXL2), and metalloproteinanses (MMPs) were included in the significantly upregulated BP processes such as ‘Cell differentiation’, ‘Cell communication’, ‘Anatomical structure morphogenesis’, and ‘Cellular developmental process’ (Fig. 3).

Among the upregulated GO MFs, ‘Cytoskeletal protein binding’ ‘Molecular function regulator’, ‘Transcription regulator activity’ and ‘Kinase activity’ were found with an upregulation of 6-Phosphofructo-2-Kinase/Fructose-2,6-Biphosphatase 3 (PFKFB3) involved in glycolysis (ESM_6). Of note, no enrichment was found in MF when comparing CTRL _FBS.

The upregulated GO CCs were significantly related to ‘Actin cytoskeleton’, ‘Anchoring junction’ in all comparisons, while in CAD _CTRL also ‘Extracellular matrix’, ‘Cell junction’ and ‘Collagen-containing extracellular matrix’ GO terms were found (ESM_7).

**Fig. 4:**
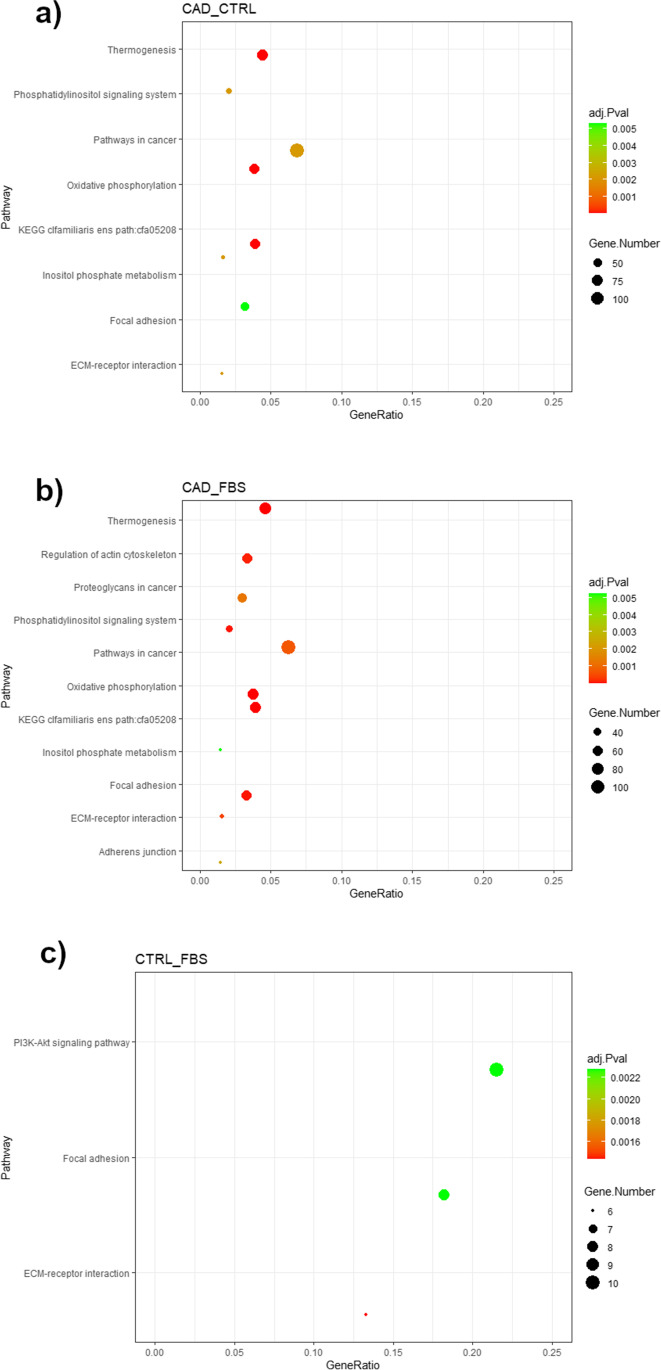
KEGG selected pathways of differentially expressed genes measured in canine dermal fibroblasts. **a** Comparison between cells incubated with serum from dogs suffering from atopic dermatitis (CAD) and with medium enriched with serum from healthy dogs (CTRL); **b** Comparison of cells incubated with serum from dogs suffering from atopic dermatitis (CAD) and with medium enriched with fetal bovine serum (FBS). **c** Comparison between cells incubated with medium enriched with serum from healthy dogs (CTRL) and with medium enriched with fetal bovine serum (FBS). The color represents the adjusted *P*-values (FDR) and the size of the spots represents the gene number. KEGG, Kyoto Encyclopedia of Genes and Genomes

**Fig. 5:**
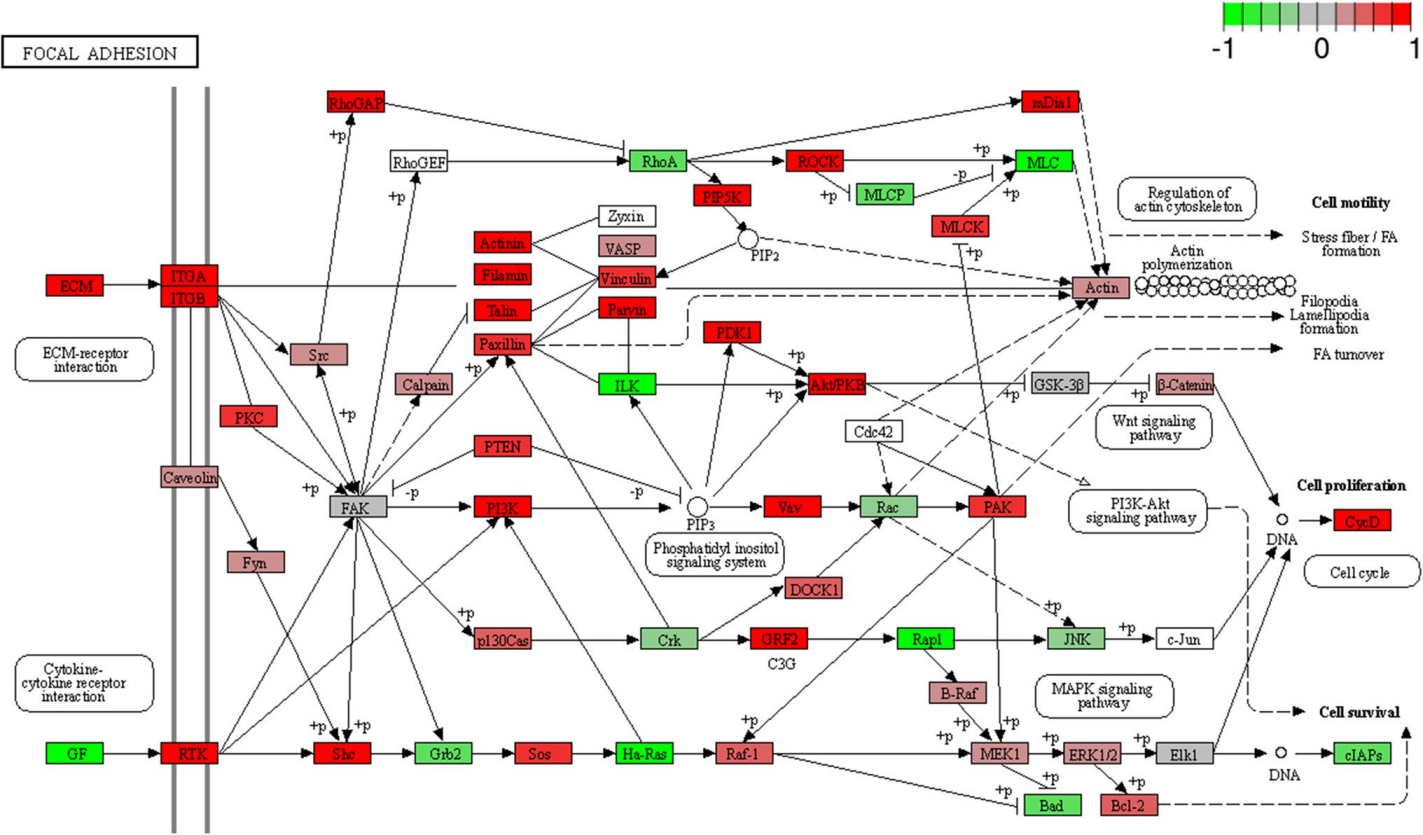
Genes differentially expressed in the KEGG pathway ‘Focal Adhesion’ measured in comparison of canine fibrobalst incubated with medium enriched in fetal bovine serum (FBS) or serum from dogs suffering from atopic dermatitis (CAD).Green: downregulated genes; Red: upregulated genes

The CAD _CTRL and CAD _FBS comparisons revealed overlapping upregulated KEGG pathways, such as ‘ECM-receptor interaction’, ‘Focal adhesion’, ‘Inositol phosphate metabolism’, with a similar number of DEGs in each pathway (ESM_8). Indeed, the CTRL _FBS comparison shared the ‘ECM receptor interaction’ and ‘Focal adhesion’ pathways with the other treatments, with very low numbers of DEGs involved (6 and 8 genes, respectively). Figure 4 shows the results of the selected KEGG enrichment in the form of a graphical representation of the scatter plots. Each panel shows selected identified pathways for each comparison with the corresponding GeneRatio, adjusted *p *value, and number of enriched genes in the corresponding pathways. The GeneRatio is defined as the number of enriched candidate genes compared to the total number of annotated genes (number of enriched genes/number of total annotated genes) considered by the KEGG analysis in the corresponding pathway. Therefore, a higher GeneRatio indicates a more significant enrichment of candidate genes in the corresponding pathway and the adjusted *p* value indicates the FDR for measurement variables of a large data set, such as for the level of gene expression of RNA sequencing data. The upregulated ‘Focal Adhesion’ KEGG pathway was reported for the CAD_FBS comparison (Fig. 5). Increased expression of integrins (ITGA2B, ITGB3) that maintain PI3K-AKT signaling was observed along with upregulation of extracellular signal-regulated kinase 1/2 (ERK1/2) of the mitogen-activated protein kinase (MAPK) pathway (Fig. 5).

Among the differentially downregulated GO BPs, ‘Mitochondrial respiratory chain complex assembly’, ‘Mitochondrion organization’ and ‘Electron transport chain’ were found significant only in CTRL _CAD and CTRL _FBS comparisons (Fig. 3 and ESM_5). The CTRL_FBS comparison showed specific downregulated terms, as ‘Adaptive immune response based on somatic recombination of immune receptors built from immunoglobulin superfamily domains’, ‘Alcohol biosynthetic process’, ‘Alcohol metabolic process’, ‘Steroid biosynthetic process’ and ‘Steroid metabolic process’.

Downregulated DEGs, among CAD_CTRL and CAD_FBS comparisons, were significantly enriched in MF, including ‘Electron transfer activity’, ‘NADH dehydrogenase activity’ and ‘RNA binding’ (ESM_6).

DEGs that specifically downregulated CCs within the comparisons CAD_CTRL and CAD_FBS were related, among others, to ‘Mitochondrial inner membrane’, ‘Mitochondrial protein complex’ and ‘Ribonucleoprotein complex’ (ESM_7).

**Fig. 6:**
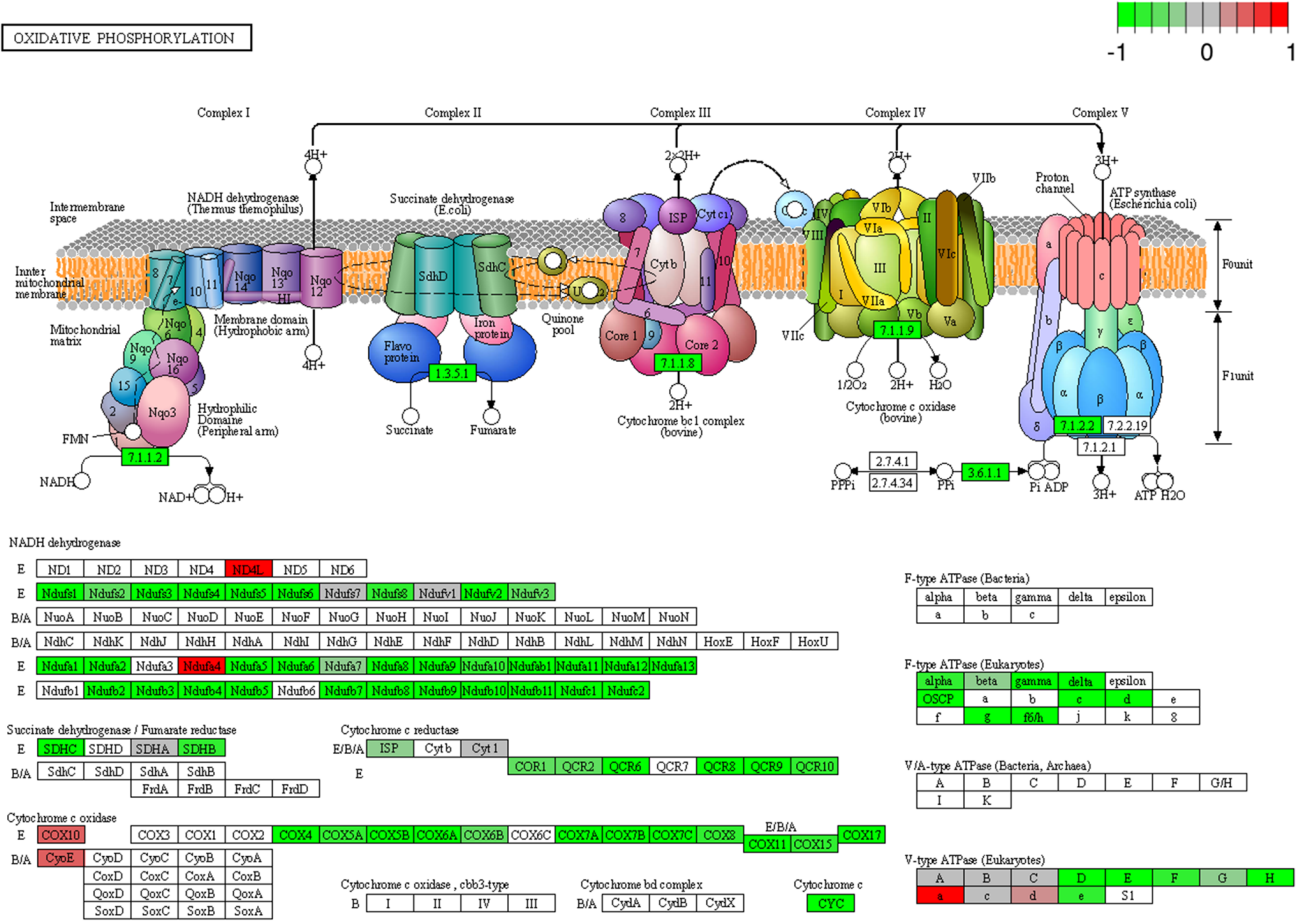
Genes differentially expressed in the KEGG pathway ‘Oxidative Phosphorylation’ measured in comparison of canine fibroblast incubated with medium enriched in fetal bovine serum (FBS) or serum from dogs suffering from atopic dermatitis (CAD). Green: downregulated genes; Red: upregulated genes

Within the KEGG pathways, several genes of the oxidative phosphorylation notably NADH dehydrogenases, succinate dehydrogenases/fumarate reductases, cytochrome C oxidases and reductases resulted downregulated (Fig. 6 and ESM_8).

## Discussion

The present study investigated the DEGs of canine dermal fibroblasts conditioned with a pool of serum sampled from healthy dogs (CTRL), or a pool of serum sampled from dogs with clinical atopic dermatitis (CAD). This approach has also been used to evaluate whether disease can affect the functions of cultured cells (Lubberich et al. 2017; Hill and Gilbert 2008; Nims and Harbell 2017).

After incubation of fibroblasts with CAD serum, stimulation of signalling pathways involved in ‘ECM-receptor interaction’, ‘Focal adhesion’ and ‘Regulation of actin cytoskeleton’, normally associated with fibroblast activation, was observed (Bergmeier et al. 2018) (ESM_8 and Fig. 4). Indeed, under normal conditions, fibroblasts increase transcription of MMP3 and decrease that of FN1 (Ghaffari et al. 2006), but during the initial phase of tissue repair and remodelling, fibroblasts adhere to the ECM, leading to focal adhesion. Fibroblast activation and phenotypic remodelling are the beginning of skin wound healing, but prolonged activation of fibroblasts can lead to excessive fibrotic responses (Pakshir et al. 2020).

When fibroblasts are activated by TGF-β1 and other specific kinases (Shroff et al. 2014), overexpression of pro-fibrotic genes occurs (Kennedy et al. 2008), leading to an increase in collagen synthesis (Ma et al. 2017; Kennedy et al. 2008). Moreover, activin A is involved in fibrotic processes and keloid formation (Morianos et al. 2019; Ham et al. 2021) by gradually inducing conversion of fibroblasts to myofibroblasts. Indeed, activin binding its receptor ActRI activates the cascade via a canonical (SMAD2/3) or non-canonical (ERK1/2) signalling that triggers a pro-fibrotic response and promotes ECM deposition and fibroblast proliferation (Vittorakis et al. 2014). Interestingly, after 24 hours of incubation with CAD, the cells showed upregulation of the activin gene (INHBA), its receptor ACVR1B, ACVR2B, and ERK1/2. In vitro response of canine skin fibroblasts, used as a model for wound healing and repair, has also shown that fibroblast activation induces MMP3 expression via inhibition of the ERK1/2 axis (Kitanaka et al. 2019). MMPs mediate ECM degradation and fibrosis resolution. In contrast, the marked downregulation of MMP3 and upregulation of COL3A1, COL5A2, COL6A1, COL6A3, COL7A1, ITGA2B, ITGA3, and FN1 observed in the present study suggests that the exposure of fibroblasts to CAD serum leads to a fibrotic phenotype. Indeed, the upregulated genes involved in the GO BP terms ‘Cell communication’, ‘Cell differentiation’ (ESM_5) and in the GO CC terms ‘Collagen-containing extracellular matrix’, ‘Actin cytoskeleton’ (ESM_7) were consistent with the transition from naïve incubated cells to activated cells observed in fibroblasts treated with CAD serum. While this property is beneficial for wound healing (Bergmeier et al. 2018), sustained activation of myofibroblasts can lead to fibrotic responses (Frantz et al. 2010). This activation has been observed in at least eight fibrotic diseases (Gu et al. 2021).

Conversely, dramatic downregulation of oxidative phosphorylation (ESM_8 and Fig. 6) and mitochondrial content was observed (Fig. 3 and ESM_7). The major downregulated genes were the supernumerary subunits of the NADH:ubiquinone oxidoreductase family (NDUFs), which are subunits of the complexes that form the mitochondrial respiratory chain. The production of ATP by glycolysis and a decrease in oxidative phosphorylation in the presence of oxygen is known as the Warburg effect or aerobic glycolysis and has been described in cancer cell lines (Liberti and Locasale 2016). This paradoxical metabolic phenotype has been described in keloid fibroblasts (Wang et al. 2021), laryngeal fibroblasts (Ma et al. 2017), tubule epithelial cells (Fierro-Fernández et al. 2020), and lung fibroblasts during pulmonary fibrosis, but not yet in skin fibroblasts during CAD. The increase in glycolysis is another sign of fibroblast transformation into myofibroblasts (Xie et al. 2015). Wang et al. (2022) suggested that upregulation of TGFB1 and the associated increase in hexokinase activity switches glucose utilisation from oxidative phosphorylation to aerobic glycolysis in renal tubule cells. Accordingly, significant upregulation of hexokinase 1 (HK1) was promoted by CAD serum in skin fibroblasts (ESM_3). Moreover, the observed activation of TGFB1/INHBA genes and downstream signalling also explained the upregulation of PFKFB3, which mediates glycolytic reprogramming in activated fibroblasts (Xie et al. 2015).

## Conclusions

The main strength of this research is to show that the fibrotic process in skin fibroblasts shares the same activation pathways that lead to upregulation of ECM deposition and downregulation of oxidative phosphorylation. To better understand the fibrotic process, a longer incubation period with CAD serum should provide more information about the molecular response of activated fibroblasts. These associated molecular signatures need to be validated in vivo and could then be used as skin biomarkers for fibrosis progression in CAD. Further research using skin fibroblasts in an ex vivo study would involve a proteomic approach to evaluate the reliability of these biomarkers in diagnosing the progression of CAD and to develop potential specific therapies.

## Supplementary Information

ESM 1 (XLSX 10 kb)

ESM 2 Modulation of MTT metabolism by serum treatments on canine dermal fibroblasts. Values are reported as mean ± standard deviation (SD) of three independent experiments (n = 12 replicates). FBS = fetal bovine serum; CTRL = pool of serum from 10 healthy dogs; CAD = pool of serum from 10 dogs with atopic dermatitis. Dermal fibroblasts were incubated for 24 hours with FBS or serum pools from dogs without (CTRL) or with (CAD) atopic dermatitis at different percentages (1.25% 2.50% 3.75% 5.00%). A nonparametric test was used to test for significant statistical differences between treatments (FBS, CTRL, CAD) within each dose. * = *P <* 0.05; *** = *P <* 0.001 differences vs. FBS (PNG 37 kb)

High Resolution Image (TIF 242 kb)

ESM 3 Volcano plots of genes measured in canine dermal fibroblasts. In red are genes that are significantly upregulated at *P* < 0.01 and have a log2 fold change greater than 1.5. In blue are genes that are significantly downregulated at *P* < 0.01 and have a log2 fold change of less than −1.5. **(a)** Comparison of gene expression levels in medium supplemented with serum from dogs with atopic dermatitis (CAD) and serum from healthy dogs (CTRL). **(b)** Comparison of gene expression levels in medium enriched with serum from dogs with atopic dermatitis (CAD) and fetal bovine serum (FBS). **(c)** Comparison of gene expression levels measured in the medium enriched with the serum from healthy dogs (CTRL) and fetal bovine serum (FBS) (PNG 621 kb)

High Resolution Image (TIF 84 kb)

ESM 4 (XLSX 1176 kb)

ESM 5 (XLSX 48 kb)

ESM 6 (XLSX 28 kb)

ESM 7 (XLSX 40 kb)

ESM 8 (XLSX 13 kb)

## Data Availability

Raw sequence data were deposited in SRA accession number: PRJNA803064 at https://www.ncbi.nlm.nih.gov/sra/PRJNA803064
