# Supplementary figures and images for: Incubation of canine dermal fibroblasts with serum from dogs with atopic dermatitis activates extracellular matrix signalling and represses oxidative phosphorylation
Source: Vet Res Commun. 2022 Jun 4;47(1):247–58. doi: 10.1007/s11259-022-09947-y (PMC9873773; doi:10.1007/s11259-022-09947-y)

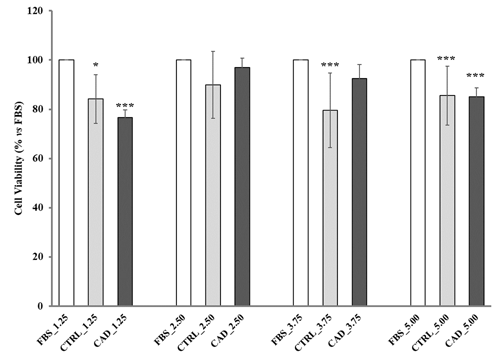

Supplement: Supplementary file 2 — Modulation of MTT metabolism by serum treatments on canine dermal fibroblasts. Values are reported as mean ± standard deviation (SD) of three independent experiments (n = 12 replicates). FBS = fetal bovine serum; CTRL = pool of serum from 10 healthy dogs; CAD = pool of serum from 10 dogs with atopic dermatitis. Dermal fibroblasts were incubated for 24 hours with FBS or serum pools from dogs without (CTRL) or with (CAD) atopic dermatitis at different percentages (1.25% 2.50% 3.75% 5.00%). A nonparametric test was used to test for significant statistical differences between treatments (FBS, CTRL, CAD) within each dose. * = P < 0.05; *** = P < 0.001 differences vs. FBS (PNG 37 kb) [file 11259_2022_9947_Fig7_ESM.png]

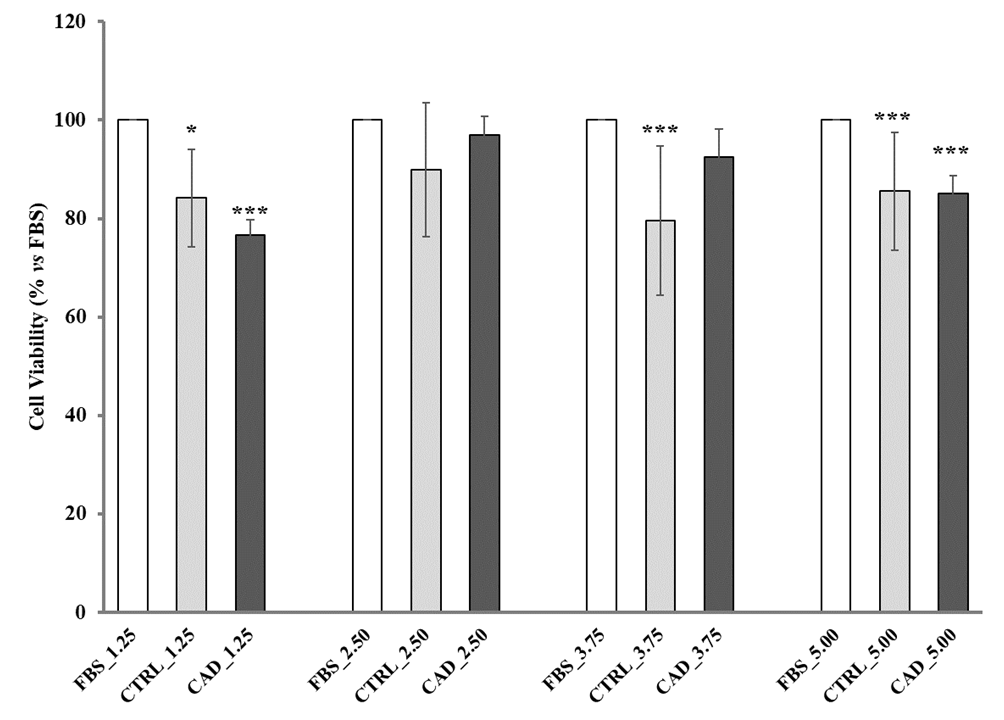

Supplement: Supplementary file 3 — High Resolution Image (TIF 242 kb) [file 11259_2022_9947_MOESM2_ESM.tif]

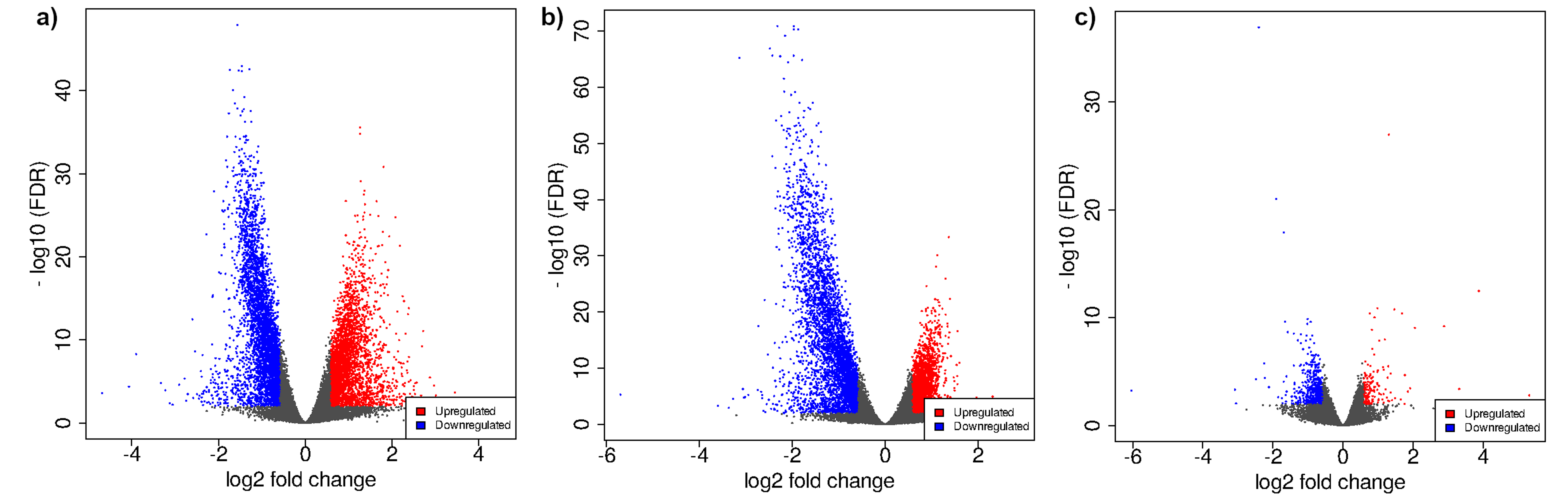

Supplement: Supplementary file 4 — Volcano plots of genes measured in canine dermal fibroblasts. In red are genes that are significantly upregulated at P < 0.01 and have a log2 fold change greater than 1.5. In blue are genes that are significantly downregulated at P < 0.01 and have a log2 fold change of less than −1.5. (a) Comparison of gene expression levels in medium supplemented with serum from dogs with atopic dermatitis (CAD) and serum from healthy dogs (CTRL). (b) Comparison of gene expression levels in medium enriched with serum from dogs with atopic dermatitis (CAD) and fetal bovine serum (FBS). (c) Comparison of gene expression levels measured in the medium enriched with the serum from healthy dogs (CTRL) and fetal bovine serum (FBS) (PNG 621 kb) [file 11259_2022_9947_Fig8_ESM.png]

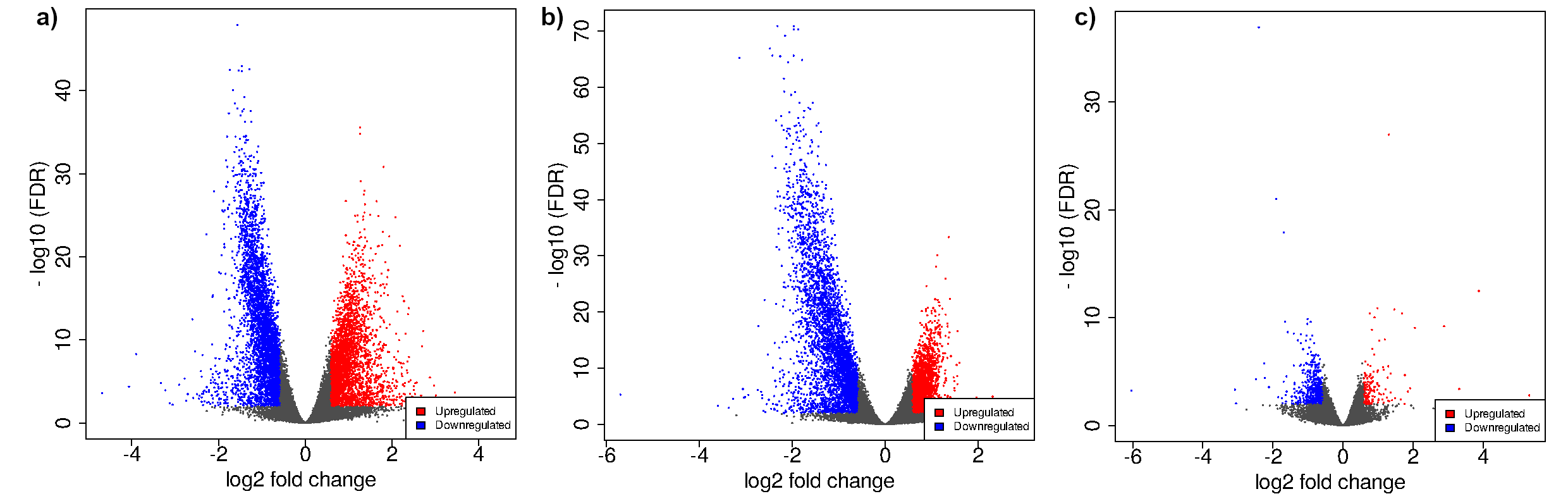

Supplement: Supplementary file 5 — High Resolution Image (TIF 84 kb) [file 11259_2022_9947_MOESM3_ESM.tif]
